# Protective Effects of *Physalis angulata* on Podocythopathies Through B-Cell-Activating Factor Inhibition in Doxorubicin-Induced Nephrotic Syndrome Rat Model

**DOI:** 10.3390/biomedicines13030719

**Published:** 2025-03-14

**Authors:** Astrid K. Kardani, Loeki E. Fitri, Nur Samsu, Krisni Subandiyah

**Affiliations:** 1 Doctoral Program in Medical Sciences, Faculty of Medicine Universitas Brawijaya, Malang 65145, East Java, Indonesia; 2 Nephrology Division, Department of Pediatric, Faculty of Medicine Universitas Brawijaya Malang 65145/Dr. Saiful Anwar General Hospital, Malang 65111, East Java, Indonesia; krisni19.fk@ub.ac.id; 3 Department of Clinical Parasitology, Faculty of Medicine Universitas Brawijaya Malang 65145/Dr. Saiful Anwar General Hospital, Malang 65111, East Java, Indonesia; 4 Nephrology Division, Department of Internal Medicine, Faculty of Medicine Universitas Brawijaya Malang 65145/Dr. Saiful Anwar General Hospital, Malang 65111, East Java, Indonesia; nur_samsu.fk@ub.ac.id

**Keywords:** nephrotic syndrome, *Physalis angulata*, BAFF, podocalyxin, nephrin, GLEPP-1, IL-4, IgG anti-nephrin, proteinuria

## Abstract

**Background/Objectives**: Nephrotic syndrome, a glomerular disease caused by podocyte dysfunction, is characterized by proteinuria, hypoalbuminemia, edema, and hyperlipidemia. Current treatment relies on corticosteroids, which carry the risk of long-term side effects. *Physalis angulata* has potential as an adjunct therapy for immune-mediated kidney injury. This study aims to evaluate the effects of *Physalis angulata* extracts on anti-nephrin IgG, IL-4, and podocytopathy through BAFF inhibition in a doxorubicin-induced nephrotic syndrome rat model. **Methods**: This experimental study involved 36 Sprague–Dawley rats divided into control and treatment groups. The treatment groups received *Physalis angulata* extract at doses of 500 mg/kgBW, 1500 mg/kgBW, and 2500 mg/kgBW, or in combination with prednisone, alongside a group receiving prednisone monotherapy. Podocytopathy was assessed using proteinuria, nephrin, podocalyxin, and GLEPP-1. Proteinuria was measured using spectrophotometry. Serum BAFF levels, renal IL-4, urinary nephrin, and urinary podocalyxin were analyzed using ELISA. Renal nephrin, renal podocalyxin, GLEPP-1, and BAFF expression were evaluated by immunofluorescence microscopy. The data were analyzed using SPSS 25. **Results**: The results showed significant reductions in proteinuria, serum BAFF levels, renal BAFF expression, anti-nephrin IgG, IL-4, urinary nephrin, and urinary podocalyxin, along with significant increases in GLEPP-1, renal nephrin, and renal podocalyxin expression, in all treatment groups compared to the nephrotic syndrome control group. The combination of *Physalis angulata* at 2500 mg/kgBW with prednisone demonstrated the best effects. **Conclusions**: *Physalis angulata* shows promise as an adjuvant therapy for nephrotic syndrome by improving podocytopathy through BAFF inhibition. Further research is needed to evaluate its long-term safety, optimize dosing, and explore clinical applications in humans.

## 1. Introduction

Nephrotic syndrome is the most common glomerular disorder in children, characterized by severe proteinuria, hypoalbuminemia, generalized oedema, and hypercholesterolemia [1]. Its incidence is estimated at 1–3 per 100,000 children, with a prevalence of 12–16 cases per 100,000 children in Southeast Asia and 6 cases per 100,000 children in Indonesia [2]. Relapse rates reach 71.9%, increasing the risk of chronic kidney disease (CKD), especially in steroid-resistant nephrotic syndrome [3].

Recent studies in nephrotic syndrome pathogenesis have shifted focus from T-cell dysregulation at the glomerular basement membrane to podocyte injury, or podocytopathy, caused by both immunologic and non-immunologic mechanisms [4]. Podocyte damage, a critical factor in maintaining the filtration barrier integrity, leads to significant proteinuria and progressive glomerulosclerosis. Persistent proteinuria is a key biomarker for diagnosis and treatment monitoring in nephrotic syndrome, with prolonged proteinuria increasing CKD risk [5,6].

Biomarkers such as nephrin and podocalyxin have emerged as sensitive indicators of podocyte injury [7,8]. Urinary podocalyxin correlates positively with proteinuria and renal dysfunction [9]. Additionally, glomerular epithelial protein 1 (GLEPP-1) is used to evaluate podocyte population, with its reduction linked to worse outcomes in lupus nephritis and IgA nephropathy [8,10].

B-cell dysregulation has also been implicated in nephrotic syndrome pathogenesis. Elevated B-cell-activating factor (BAFF) levels in nephrotic syndrome have been associated with worse renal outcomes [3]. BAFF plays a crucial role in B-cell maturation and differentiation, with its dysregulation potentially contributing to podocyte injury through immune-mediated mechanisms [11]. Targeted therapies, such as rituximab, have shown promise but are associated with severe complications like neutropenia-related infections [12].

Given the limitations of current therapies, interest in plant-based treatments has grown. *Physalis angulata* (ciplukan), a plant native to Indonesia, contains bioactive compounds such as withanolides, exhibiting potent immunomodulatory effects. These compounds have demonstrated anti-inflammatory and renal-protective properties in animal models of immune-mediated diseases [13,14]. Despite its potential, no studies have evaluated the effects of *Physalis angulata* in nephrotic syndrome.

This study aims to investigate the impact of *Physalis angulata* extract on proteinuria and podocyte injury in a nephrotic syndrome rat model, focusing on its potential to modulate BAFF signaling. Findings may offer insights into alternative therapeutic approaches for managing nephrotic syndrome.

## 2. Materials and Methods

### 2.1. Study Design

This study was conducted using a true experimental post-test-only controlled group design, with rats used as animal models and randomly assigned to groups.

### 2.2. Physalis angulata Extraction

*Physalis angulata* leaves were harvested from Sidomulyo Village, Batu City, East Java, Indonesia. *Physalis angulata* leaves were extracted using the maceration method. *Physalis angulata* leaf powder was macerated with 96% ethanol solvent. HPLC analysis was carried out based on the method used by Mastuti et al. (2019) in previous research [15]. The extract concentrations (500 mg/KgBW, 1500 mg/KgBW, and 2500 mg/KgBW) were obtained from the results of previous studies examining *Physalis angulata* [16,17,18].

### 2.3. Animal Model

All research procedures were ethically approved by the Research Ethics Committee of Universitas Brawijaya under registration number 050-KEP-UB-2024. Male Sprague–Dawley rats, aged 8 weeks and weighing 200 ± 20 g, were obtained from Biomedical Technology Indonesia, Bogor, Indonesia. The rats were acclimatized in the Laboratory of Experimental Animal Development at the Faculty of Medicine, Universitas Brawijaya, for 7 days before the experiment. The rats were acclimatized in the Laboratory of Experimental Animal Development at the Faculty of Medicine, Universitas Brawijaya, for 7 days before the experiment. They were provided ad libitum access to a standard PARS rat diet and water. The rats were housed under standard conditions of temperature (22 ± 3 °C), relative humidity (55 ± 10%), and a 12:12 h light–dark cycle. Urinalysis was conducted for initial screening to exclude rats that already had proteinuria by utilizing Siemens Multistix 8 SG Urine Reagent Test Strips, acquired from the clinical pathology laboratory of the Faculty of Medicine, Universitas Brawijaya.

A nephrotic syndrome model in rats was induced using a single intravenous injection of doxorubicin, obtained from Global Onkolab Farma (Kalbe Farma, Indonesia), at a dose of 7 mg/kg body weight, a measure which refers to our preliminary study. The rats were placed in a restrainer to ensure immobilization before injection. Their tails were immersed in warm water to dilate the tail veins. The tails were then dried and cleaned with 70% alcohol swabs. The tail veins were immobilized, and the target vein for injection was identified. Once the vein was located, doxorubicin was injected into the vein using a prepared 24G intravenous catheter. The negative control group received an intravenous injection of 1 mL of normal saline without doxorubicin. The induction of nephrotic syndrome was confirmed six weeks after the administration of doxorubicin [19,20].

Animal models were divided into 9 treatment groups. The minimum sample size was calculated using the Federer Formula t(n − 1) > 15, where n is the number of samples per treatment group and t is the number of treatment groups. Three groups received single administrations of *Physalis angulata* extract at doses of 500 mg/KgBW (PA 500), 1500 mg/KgBW (PA 1500), and 2500 mg/KgBW (PA 2500), 3 groups received *Physalis angulata* of each dose + prednisone therapy with an animal equivalent dose of 12.4 mg/Kg, 1 group received only prednisone, 1 group of healthy rats was a negative control, and there was 1 group of nephrotic syndrome model rats without any treatment. The treatment was administered once daily for 21 days.

### 2.4. Measurement of Proteinuria and Biochemical Markers

To quantify the 24-h urinary protein levels, urine specimens were collected using metabolic cages. Subsequently, the urine samples underwent centrifugation at 10,000 rpm for 15 min at 4 °C. Post-centrifugation, the specimens were supplemented with bovine serum albumin (BSA) as a buffering agent. The absorbance readings were obtained at a wavelength of 595 nm using the Bio-Rad iMark™ Microplate Absorbance Reader from Bio-Rad (Hercules, CA, USA)

Rats were anaesthetized using ketamine before blood samples were taken from the heart. Blood samples were obtained through cardiac dissection and stored in serum separator tubes (SST) for subsequent centrifugation at 5000 rpm at 4 °C for 15 min. The supernatant was then collected for the measurement of albumin (serum albumin assay kit, Cat No.: A028-2-1 from Nanjing Jincheng Institute of Biotechnology (Nanjing, China)) and cholesterol levels (total cholesterol assay kit, Cat No.: A111-1-1 from Nanjing Jincheng Institute of Biotechnology (Nanjing, China)) using an autoanalyzer.

### 2.5. Enzyme-Linked Immunosorbent Assay (ELISA)

Antinephrin IgG and BAFF from serum, IL-4 in the kidney, urinary nephrin and podocalyxin were measured by ELISA. ELISA kit used in this study including BAFF ELISA kit from Assay genie ((Wicklow, Ireland; Product No.: RTDL00130)), nephrin ELISA kit from Elabscience (Wuhan, China; Product No.: E-EL-R2406), podocalyxin ELISA kit from Assay genie (Wicklow, Ireland; Product No.: RTFI01034), Rat IgG anti-nephrin from My BioSource (San Diego, CA, USA; Product No.: MBS7276112), and IL-4 ELISA kit from Elabscience (Wuhan, China; Product No.: E-EL-R0014). The protocol was carried out following the manufacturer’s instructions.

### 2.6. Immunoflourescence

The formalin-fixed, paraffin-embedded (FPPE) kidney was sectioned (3 μm thick). Expression of BAFF, nephrin, podocalyxin, and GLEPP-1 was measured by Immunofluorescence. Rabbit anti-BAFF primary antibody BAFF from Bioss (Beijing, China; Product No.: bs-2431R), mouse nephrin antibody from SCBT (Dallas, TX, USA; Product No.: sc-376522), podocalyxin rabbit antibody from Bioss (Beijing, China; Product No.: bs-1345R), and mouse GLEPP1 polyclonal antibody from Bioss (Santa Cruz Biotechnology, Dallas, TX, USA; Product No.: sc-365354) were used as the primary antibody.

The deparafinization process was carried out starting with the slide being heated at 600 °C for 60 min. The samples were then rehydrated by soaking in: xylol solution (2 × 10 min), absolute ethanol (2 × 10 min), 95% ethanol (1 × 5 min), 70% ethanol (1 × 5 min), 50% ethanol (1 × 5 min). The slides were then washed with deionized H_2_O. Slides were rehydrated with a wash buffer for 10 min. Excess wash buffer is then discarded. Afterwards, the protocol was carried out following the manufacturer’s instructions. The reading is immediately performed using Olympus IX71. We quantified approximately 20–30 glomeruli per sample. The data was quantified using ImageJ version 1.53 (https://imagej.net/ij/, accessed on 15 September 2024).

### 2.7. Data Analysis

The data analysis was performed using the Statistical Package for the Social Sciences (SPSS) software, 25th version. The Kruskal–Wallis test was used to compare the significance across all treatment groups with the negative control group. Post hoc analysis was conducted using the Mann–Whitney test.

## 3. Results

### 3.1. Serum and Kidney BAFF Levels

**Figure 1:**
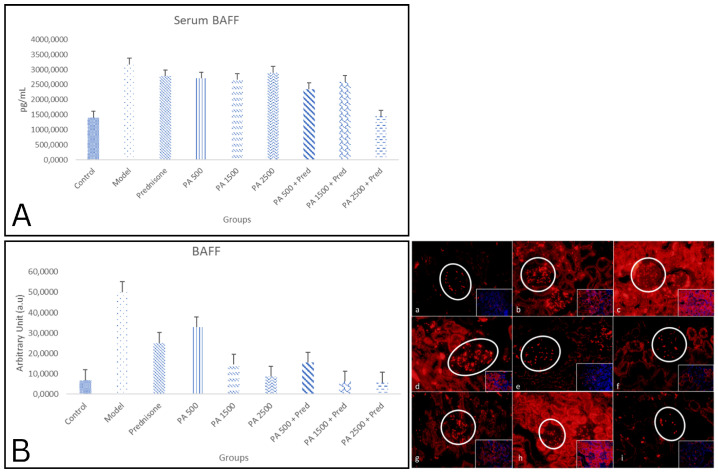
(**A**) A histogram of serum BAFF levels in the study groups. *p* < 0.05 was found in control vs. model; model vs. PA 1500 + prednisone; and model vs. PA 2500 + prednisone groups. (**B**) A histogram of kidney BAFF expression in the study groups. *p* < 0.05 found in control vs. model; control vs. prednisone; control vs. PA 500; control vs. PA 1500; model vs. prednisone; model vs. PA 500; model vs. PA 1500; model vs. PA 2500; model vs. PA 500 + pred; model vs. PA 1500 + prednisone; and model vs. PA 2500 + prednisone groups; prednisone vs. PA 2500; prednisone vs. PA 1500 + prednisone; prednisone vs. PA 2500 + prednisone. The expression of BAFF in kidneys is shown in Figure (**a**–**i**). BAFF immunofluorescence in kidneys at 400× magnification. (**a**) Negative control; (**b**) nephrotic syndrome model; (**c**) prednisone; (**d**) *Physalis angulata* at a dose of 500 mg/kgBW; (**e**) *Physalis angulata* at a dose of 1500 mg/kgBW; (**f**) *Physalis angulata* at a dose of 2500 mg/kgBW; (**g**) *Physalis angulata* at a dose of 500 mg/kgBW + prednisone; (**h**) *Physalis angulata* at a dose of 1500 mg/kgBW + prednisone; (**i**) *Physalis angulata* at a dose of 2500 mg/kgBW + prednisone. White circles indicate BAFF expression in the glomerulus. The image in the lower right corner represents a composite view of the antibody and DAPI.

The results of this study showed a significant difference in serum BAFF levels between the negative control group and the nephrotic syndrome model group, as shown in Figure 1A. In the nephrotic syndrome model group, serum BAFF levels were significantly increased compared to the negative control group. In the treatment groups, a significant decrease in serum BAFF levels was observed in the *Physalis angulata* 2500 mg/kgBW + prednisone group compared to the nephrotic syndrome model group. The BAFF levels in the *Physalis angulata* 2500 mg/kgBW + prednisone group remained higher than in the negative control group, but the difference was not significant. Meanwhile, in the other *Physalis angulata* groups and *Physalis angulata* + prednisone groups, serum BAFF levels did not differ significantly compared to the nephrotic syndrome model group and were significantly higher compared to the negative control group. This was also observed in the group receiving prednisone alone. These findings indicate that the combination of *Physalis angulata* at a dose of 2500 mg/kgBW and prednisone provides the best effect on improving proteinuria conditions in nephrotic syndrome model rats (*p* < 0.05).

This study also showed a significant difference in renal BAFF expression between the negative control group and the nephrotic syndrome model group. Figure 1B shows a comparison of the immunofluorescence results for BAFF in the kidneys of healthy rats and nephrotic syndrome model rats. In this study, BAFF expression was measured in the cytoplasm, excluding the nucleus.

In the nephrotic syndrome model group, kidney BAFF expression was significantly increased compared to the negative control group, as shown in Figure 1. A significant decrease in renal BAFF expression was observed in all treatment groups, whether treated with prednisone, *Physalis angulata*, or *Physalis angulata* + prednisone, compared to the nephrotic syndrome model group. Renal BAFF expression in the *Physalis angulata* 2500 mg/kgBW, *Physalis angulata* 1500 mg/kgBW + prednisone, and *Physalis angulata* 2500 mg/kgBW + prednisone groups was significantly lower compared to the prednisone group. These findings indicate that the administration of *Physalis angulata* at 1500 and 2500 mg/kgBW can reduce renal BAFF expression compared to the prednisone-only group.

### 3.2. IL-4 and IgG Anti-Nephrin

**Figure 2:**
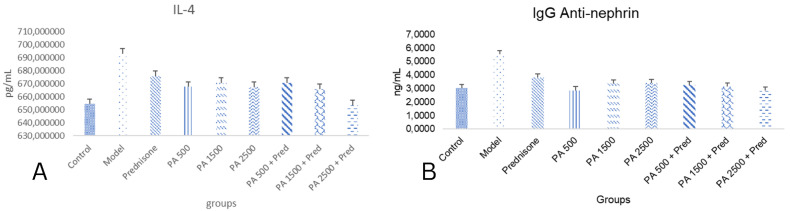
(**A**) A histogram of kidney IL-4 (pg/mL tissue) and anti-nephrin IgG levels in the study groups. *p* < 0.05 was found in control vs. model; control vs. prednisone; model vs. PA 500; model vs. PA 1500; model vs. PA 2500; model vs. + PA 500 + prednisone; model vs. PA 2500 + prednisone groups. (**B**) A histogram of serum IgG anti-nephrin levels in the study groups. *p* < 0.05 found in control vs. model; model vs. PA 500; model vs. PA 1500; model vs. PA 2500; model vs. PA 500 + prednisone; model vs. PA 1500+ prednisone; model vs. PA 2500 + prednisone groups.

The results of this study demonstrated a significant difference in renal IL-4 levels between the negative control group and the nephrotic syndrome model group (*p* < 0.05), as shown in Figure 2. In the nephrotic syndrome model group, renal IL-4 levels were significantly increased compared to the negative control group. Renal IL-4 levels in all treatment groups, including prednisone, *Physalis angulata*, and *Physalis angulata* + prednisone, were significantly decreased compared to the nephrotic syndrome model group. The greatest reduction in renal IL-4 levels was observed in the *Physalis angulata* 1500 mg/kgBW + prednisone and *Physalis angulata* 2500 mg/kgBW + prednisone groups. The IL-4 levels in the *Physalis angulata* 2500 mg/kgBW + prednisone group were significantly lower than in the prednisone-only group. These findings indicate that *Physalis angulata* at a dose of 2500 mg/kgBW can reduce renal IL-4 levels in nephrotic syndrome model rats, and its combination with prednisone provides a superior effect.

The results of this study demonstrated a significant difference in anti-nephrin IgG levels between the negative control group and the nephrotic syndrome model group (*p* < 0.05), as shown in Figure 2B. In the nephrotic syndrome model group, anti-nephrin IgG levels were significantly increased compared to the negative control group. A significant reduction in anti-nephrin IgG levels was observed in all treatment groups, including prednisone, *Physalis angulata*, and *Physalis angulata* + prednisone, compared to the nephrotic syndrome model group. In the *Physalis angulata* 500 mg/kgBW and *Physalis angulata* 2500 mg/kgBW + prednisone groups, anti-nephrin IgG levels were significantly lower compared to the prednisone-only group. These findings indicate that *Physalis angulata* at a dose of 2500 mg/kgBW can reduce anti-nephrin IgG levels more effectively than prednisone alone.

### 3.3. Reduction in Proteinuria

**Figure 3:**
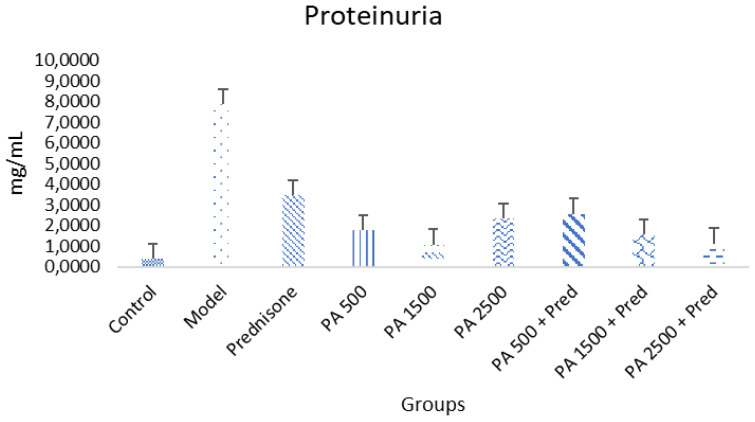
A histogram of serum proteinuria in the study groups. *p* < 0.05 was found in control vs. model; control vs. prednisone; control vs. PA 500; control vs. PA 1500; control vs. PA 2500; control vs. PA 500 + prednisone; control vs. PA 1500 + prednisone; control vs. PA 1500 + prednisone; model vs. prednisone; model vs. PA 500; model vs. PA 1500; model vs. PA 2500; model vs. PA 500 + prednisone; model vs. PA 1500+ prednisone; model vs. PA 2500 + prednisone groups.

The results of this study showed a significant difference in proteinuria levels between the negative control group and the nephrotic syndrome model group (*p* < 0.05), as shown in Figure 3. In the nephrotic syndrome model group, proteinuria levels were significantly higher compared to the negative control group. Conversely, a significant reduction in proteinuria levels was observed in all treatment groups, including the prednisone-only group, the *Physalis angulata*-only group, and the *Physalis angulata* + prednisone combination group, compared to the nephrotic syndrome model group. However, proteinuria levels in all treatment groups remained higher than those in the negative control group. This indicates that although the administration of *Physalis angulata* can reduce proteinuria levels, the levels have not yet returned to normal as observed in the negative control group. Additionally, significant differences were found between the groups receiving *Physalis angulata* at a dose of 500 mg/kgBW, *Physalis angulata* at a dose of 1500 mg/kgBW + prednisone, and *Physalis angulata* at a dose of 2500 mg/kgBW + prednisone compared to the prednisone-only group.

### 3.4. Podocytopathy Markers

#### 3.4.1. Nephrin

**Figure 4:**
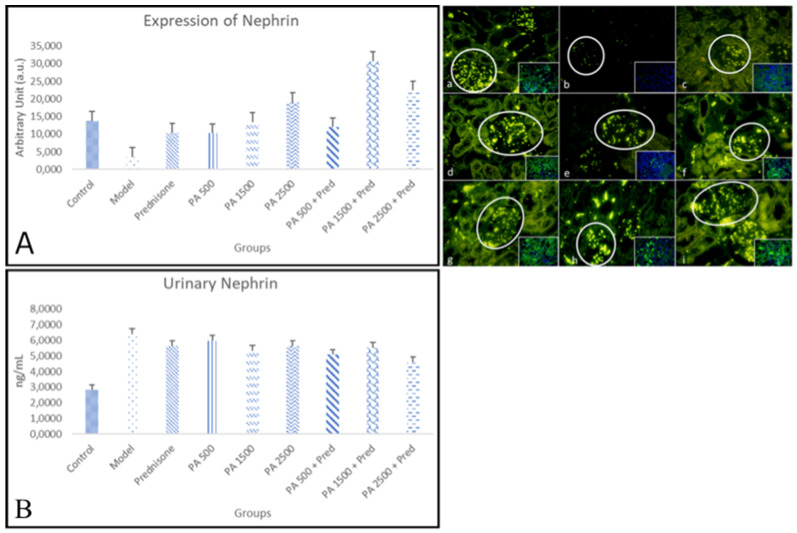
The expression of nephrin in kidneys is shown in Figure (**a**–**i**). Nephrin immunofluorescence in kidneys at 400× magnification. (**a**) Negative control; (**b**) nephrotic syndrome model; (**c**) prednisone; (**d**) *Physalis angulata* at a dose of 500 mg/kgBW; (**e**) *Physalis angulata* at a dose of 1500 mg/kgBW; (**f**) *Physalis angulata* at a dose of 2500 mg/kgBW; (**g**) *Physalis angulata* at a dose of 500 mg/kgBW + prednisone; (**h**) *Physalis angulata* at a dose of 1500 mg/kgBW + prednisone; (**i**) *Physalis angulata* at a dose of 2500 mg/kgBW + prednisone. White circles indicate nephrin expression in the glomerulus. The image in the lower right corner represents a composite view of the antibody and DAPI. (**A**) A histogram of nephrin expression in the study groups. A histogram of nephrin levels in the study groups. *p* < 0.05 was found in control vs. model; control vs. PA 1500 + prednisone; model vs. PA 1500; model vs. PA 2500; model vs. PA 500 + prednisone model vs. PA 1500 + prednisone; and model vs. PA 2500 + prednisone groups. (**B**) A histogram of urinary nephrin levels in the study groups. *p* < 0.05 was found in control vs. model; control vs. prednisone; control vs. PA 500; control vs. PA 1500; control vs. PA 2500; control vs. PA 500 + prednisone; control vs. PA 1500 + prednisone; model vs. prednisone; PA 500; model vs. PA 1500; model vs. PA 2500; model vs. PA 500 + prednisone; model vs. PA 1500 + prednisone; and model vs. PA 2500 + prednisone groups.

Figure 4A shows a comparison of the immunofluorescence results for nephrin in the kidneys of healthy rats and nephrotic syndrome model rats. In this study, nephrin expression was measured in the cytoplasm, excluding the nucleus.

The results of this study showed a significant difference in renal nephrin expression between the negative control group and the nephrotic syndrome model group as shown in Figure 4. In the nephrotic syndrome model group, renal nephrin expression was significantly reduced compared to the negative control group. Nephrin expression in the *Physalis angulata* 1500 mg/kgBW, *Physalis angulata* 2500 mg/kgBW, and all *Physalis angulata* + prednisone groups were higher compared to the nephrotic syndrome model group. The highest renal nephrin expression was observed in the *Physalis angulata* 1500 mg/kgBW + prednisone group, which was significantly higher than the prednisone group. This indicates that the administration of *Physalis angulata* can prevent the reduction of nephrin expression in the kidneys of nephrotic syndrome model rats, and the combination with prednisone provides a better effect.

The study also showed a significant difference in urinary nephrin levels between the negative control group and the nephrotic syndrome model group (*p* < 0.05) as shown in Figure 4B. In the nephrotic syndrome model group, urinary nephrin levels were significantly increased compared to the negative control group. Urinary nephrin levels in all treatment groups, including prednisone, *Physalis angulata*, and *Physalis angulata* + prednisone, were significantly reduced compared to the nephrotic syndrome model group. The greatest reduction in urinary nephrin levels was observed in the *Physalis angulata* 2500 mg/kgBW + prednisone group. The urinary nephrin levels in this group were significantly lower compared to the prednisone-only group. This indicates that the administration of *Physalis angulata* at a dose of 2500 mg/kgBW can reduce urinary nephrin levels in nephrotic syndrome model rats, and the combination with prednisone provides a better effect.

#### 3.4.2. Podocalyxin

**Figure 5:**
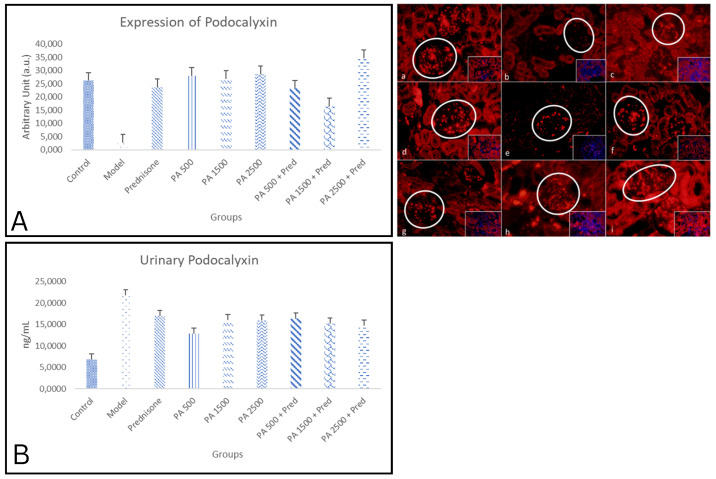
The expression of podocalyxin in kidneys is shown in Figure (**a**–**i**). Podocalyxin immunofluorescence in kidneys at 400× magnification. (**a**) Negative control; (**b**) nephrotic syndrome model; (**c**) prednisone; (**d**) *Physalis angulata* at a dose of 500 mg/kgBW; (**e**) *Physalis angulata* at a dose of 1500 mg/kgBW; (**f**) *Physalis angulata* at a dose of 2500 mg/kgBW; (**g**) *Physalis angulata* at a dose of 500 mg/kgBW + prednisone; (**h**) *Physalis angulata* at a dose of 1500 mg/kgBW + prednisone; (**i**) *Physalis angulata* at a dose of 2500 mg/kgBW + prednisone. White circles indicate nephrin expression in the glomerulus. The image in the lower right corner represents a composite view of the antibody and DAPI. (**A**) A histogram of podocalyxin levels in the study groups. *p* < 0.05 was found in control vs. model; model vs. prednisone; prednisone vs. PA 500; prednisone vs. PA 1500; prednisone vs. PA 1500; prednisone vs. pa 500 + prednisone; prednisone vs. PA 2500 + prednisone; model vs. PA 500; model vs. PA 1500; model vs. PA 2500; model vs. PA 500 + prednisone; model vs. PA 1500 + prednisone; and model vs. PA 2500 + prednisone groups. (**B**) A histogram of urinary podcalyxin levels in the study groups. *p* < 0.05 was found in control vs. model; control vs. prednisone; control vs. PA 500; control vs. PA 1500; control vs. PA 2500; control vs. PA 500 + prednisone; control vs. PA 1500 + prednisone; model vs. prednisone; PA 500; model vs. PA 1500; model vs. PA 2500; model vs. PA 500 + prednisone; model vs. PA 1500 + prednisone; and model vs. PA 2500 + prednisone groups.

Figure 5 shows a comparison of the immunofluorescence results for podocalyxin in the kidneys of healthy rats and nephrotic syndrome model rats. Podocalyxin expression was measured in the cytoplasm, excluding the nucleus.

The study showed a significant difference in renal podocalyxin expression between the negative control group and the nephrotic syndrome model group (*p* < 0.05) as shown in Figure 5A. In the nephrotic syndrome model group, renal podocalyxin expression was significantly decreased compared to the negative control group. Renal podocalyxin expression in all treatment groups, including prednisone, *Physalis angulata*, and *Physalis angulata* + prednisone, was significantly higher than in the nephrotic syndrome model group. Podocalyxin expression in the *Physalis angulata* groups was higher than in the prednisone group, although the difference was not statistically significant. The highest renal podocalyxin expression was observed in the *Physalis angulata* 2500 mg/kgBW + prednisone group, which was significantly higher compared to the prednisone-only group. This indicates that the administration of *Physalis angulata* at a dose of 2500 mg/kgBW can prevent the decline in podocalyxin expression in the kidneys of nephrotic syndrome model rats, and the combination with prednisone provides a better effect.

A significant difference in urinary podocalyxin levels also found between the negative control group and the nephrotic syndrome model group as shown in Figure 5B. In the nephrotic syndrome model group, urinary podocalyxin levels were significantly increased compared to the negative control group. Urinary podocalyxin levels in all treatment groups, including prednisone, *Physalis angulata*, and *Physalis angulata* + prednisone, decreased compared to the nephrotic syndrome model group. However, the decrease was not statistically significant, with the greatest reduction observed in the *Physalis angulata* 2500 mg/kgBW group.

#### 3.4.3. GLEPP-1

**Figure 6:**
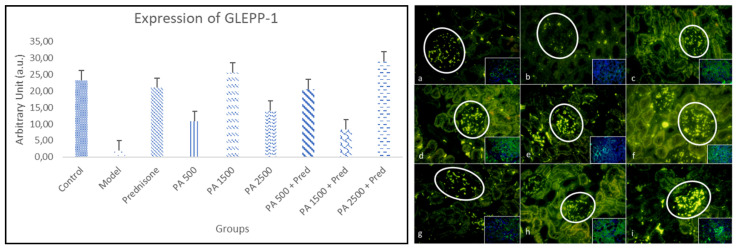
The expression of GLEPP-1 in kidneys is shown in Figure (**a**–**i**). GLEPP-1 immunofluorescence in kidneys at 400× magnification. (**a**) Negative control; (**b**) nephrotic syndrome model; (**c**) prednisone; (**d**) *Physalis angulata* at a dose of 500 mg/kgBW; (**e**) *Physalis angulata* at a dose of 1500 mg/kgBW; (**f**) *Physalis angulata* at a dose of 2500 mg/kgBW; (**g**) *Physalis angulata* at a dose of 500 mg/kgBW + prednisone; (**h**) *Physalis angulata* at a dose of 1500 mg/kgBW + prednisone; (**i**) *Physalis angulata* at a dose of 2500 mg/kgBW + prednisone. White circles indicate nephrin expression in the glomerulus. The image in the lower right corner represents a composite view of the antibody and DAPI. The histogram of GLEPP-1 expression in the study groups. *p* < 0.05 was found in control vs. model; control vs. PA 1500 + prednisone; model vs. prednisone; model vs. PA 500; model vs. PA 1500; model vs. PA 2500; model vs. PA 500 + prednisone; model vs. PA 1500 + prednisone; and model vs. PA 2500 + prednisone groups.

Figure 6 shows a comparison of the immunofluorescence results for GLEPP-1 in the kidneys of healthy rats and nephrotic syndrome model rats. GLEPP-1 expression was measured in the cytoplasm, excluding the nucleus.

The results of this study demonstrated a significant difference in GLEPP-1 expression between the negative control group and the nephrotic syndrome model group (*p* < 0.05), as shown in Figure 6. In the nephrotic syndrome model group, GLEPP-1 expression was significantly decreased compared to the negative control group. GLEPP-1 expression in all treatment groups, including prednisone, *Physalis angulata*, and *Physalis angulata* + prednisone, was significantly higher than in the nephrotic syndrome model group. GLEPP-1 expression in all groups receiving *Physalis angulata* was higher compared to the prednisone-only group; however, significant differences were observed only in the *Physalis angulata* 1500 mg/kgBW and *Physalis angulata* 2500 mg/kgBW + prednisone groups. The highest GLEPP-1 expression was found in the *Physalis angulata* 2500 mg/kgBW + prednisone group. This indicates that *Physalis angulata* at doses of 1500 and 2500 mg/kgBW can increase GLEPP-1 expression compared to the prednisone-only group (*p* < 0.05).

## 4. Discussion

### 4.1. Role of BAFF Inhibition

The results of this study demonstrate a significant increase in serum BAFF levels in the nephrotic syndrome model group, supporting the hypothesis regarding the role of dysregulated B-cell activation mediated by its activator, BAFF, in the pathogenesis of nephrotic syndrome [21,22,23,24]. The rise in serum BAFF levels in nephrotic syndrome is a response to TLR activation triggered by various initiating factors [25,26]. Toll-like receptors, part of the innate immune system, recognize structural components of microorganisms through the detection of danger-associated molecular patterns (DAMPs) and pathogen-associated molecular patterns (PAMPs), initiating an immediate pro-inflammatory response. TLR activation triggers the innate immune response, leading to the cleavage of BAFF attached to myeloid cells (the primary BAFF producers) by furin protease, producing soluble BAFF. Additionally, TLR activation further increases BAFF expression [27,28,29].

This study also investigated BAFF expression in the kidneys of nephrotic syndrome model rats. Locally, BAFF is expressed by renal tubular cells and is implicated in the pathophysiology of lupus nephritis by allowing autoreactive B cells to escape peripheral tolerance [30]. BAFF expression in the kidney provides a more representative indicator of severity and therapeutic response in lupus nephritis compared to serum BAFF expression. Increased BAFF expression in the kidney induces an inflammatory response by activating B cells, which release pro-inflammatory cytokines like IL-4, while simultaneously recruiting more inflammatory cells to the glomeruli, exacerbating podocyte damage [31].

The significant increase in BAFF expression in the kidneys of nephrotic syndrome model rats compared to the negative control group aligns with previous research. Their study found elevated BAFF expression in the podocytes of pediatric idiopathic nephrotic syndrome patients, associated with decreased renal function [3].

The role of BAFF in nephrotic syndrome emphasizes its function as a key mediator, particularly in promoting harmful humoral immune responses to podocytes. Targeting BAFF therapeutically could effectively disrupt the inflammatory cycle and protect podocyte structures. This study underscores the importance of developing BAFF-modulating therapies for more effective nephrotic syndrome management.

The reduction in serum BAFF levels in the treatment groups indicates the role of *Physalis angulata* in BAFF inhibition. *Physalis angulata* is known to contain withanolides, which not only reduce oxidative stress-induced free radicals but also exhibit immunomodulatory effects by suppressing immune cell activation, particularly B cells [32,33,34]. The immunomodulatory effects of withanolides suppress immune cascades involved in the pathogenesis of nephrotic syndrome, including glomerular damage and podocytopathy [23,35,36].

In previous in silico studies, withanolides demonstrated direct inhibitory effects on BAFF and its receptor, BCMA. Additionally, another bioactive component of *Physalis angulata*, physalin F, inhibited another BAFF receptor, TACI [34]. In this study, significant reductions in serum BAFF levels were observed in the *Physalis angulata* 1500 mg/kgBW + prednisone and *Physalis angulata* 2500 mg/kgBW + prednisone groups compared to both the nephrotic syndrome model group and the prednisone-only group. This indicates a potential synergistic effect between *Physalis angulata* and prednisone. When co-administered with methylprednisolone, *Physalis angulata* extract reduced inflammation and improved renal function in lupus model rats by mitigating immune responses and reducing apoptosis caused by immune response-induced oxidative stress [13]. The transcription factor activity of glucocorticoid receptors on BAFF may also contribute to BAFF inhibition through the phytosteroid effects of *Physalis angulata* [14].

Similar to the mechanism of serum BAFF reduction, decreased BAFF expression in the kidney is mediated by BAFF inhibition due to the phytosteroid effects of withanolides and physalin F in *Physalis angulata* [14]. BAFF activation in renal tissue is known to trigger lymphocyte activity, recruiting inflammatory cytokines that cause podocyte damage. BAFF inhibition reduces immune cell activity and prevents podocyte damage from cytokines locally produced by renal B cells [33,37].

Currently, no studies have examined the relationship between serum BAFF levels and renal BAFF expression in nephrotic syndrome. However, this study shows a significant positive correlation between serum BAFF levels and renal BAFF expression. The TLR-mediated activation of the innate immune system triggers immune cascades, including increased BAFF levels, which activate B cells. This immune activation allows immune cell infiltration into the kidney. Infiltrated immune cells, such as macrophages and dendritic cells, along with resident renal cells, express BAFF, further activating lymphocytes in the kidney [28,37].

### 4.2. Effects of Physalis angulata in IL-4 and Anti-Nephrin IgG

This study demonstrates that the administration of *Physalis angulata* in a nephrotic syndrome model leads to a significant reduction in both IgG anti-nephrin and IL-4 levels compared to the nephrotic syndrome model group without treatment. The greatest decrease was observed in the group receiving the combination of *Physalis angulata* at a dose of 2500 mg/KgBB with prednisone. The increase in IgG anti-nephrin levels in the nephrotic syndrome model group supports the hypothesis that autoantibodies, particularly IgG anti-nephrin targeting nephrin on the slit diaphragm, may be involved in the pathogenesis of nephrotic syndrome. The reduction in IgG anti-nephrin after treatment with *Physalis angulata* indicates the immunoregulatory effect of this plant, which modulates B-cell activity.

B-cell-activating factor (BAFF) plays an important role in B-cell maturation and antibody production, including pathogenic antibodies such as IgG anti-nephrin. The reduction in BAFF induced by *Physalis angulata* may decrease B-cell activity, including the production of autoantibodies that exacerbate proteinuria and renal inflammation, as seen in lupus nephritis patients [37,38]. The reduction in IgG anti-nephrin in the group treated with the combination of *Physalis angulata* and prednisone also suggests there is a synergistic effect between the two therapies, where *Physalis angulata* enhances the effectiveness of prednisone as the primary therapy for nephrotic syndrome.

Furthermore, the administration of *Physalis angulata* also affected the renal IL-4 levels, which were significantly elevated in the nephrotic syndrome model group without treatment. IL-4, produced by activated B cells, is known to contribute to proteinuria through antibody-independent mechanisms [39,40]. The reduction in IL-4 levels in the treatment groups suggests that *Physalis angulata*, through its immunomodulatory effects, inhibits B-cell activation both systemically and locally, reducing the production of inflammatory cytokines like IL-4, which contribute to renal inflammation [14,29,39,41]. Thus, *Physalis angulata* shows potential as an adjunctive therapy that can reduce the need for high doses of prednisone, improve therapeutic effectiveness, and decrease the risk of long-term side effects in nephrotic syndrome patients.

Overall, these findings provide additional evidence for the potential of *Physalis angulata* in managing nephrotic syndrome through the modulation of the immune response, specifically in regulating autoantibody production and inflammatory cytokine levels, which contribute to renal improvement in nephrotic syndrome patients.

### 4.3. Proteinuria

The results of this study demonstrate the effect of *Physalis angulata* extract in reducing proteinuria levels in nephrotic syndrome model rats. The significant increase in proteinuria observed in the nephrotic syndrome model group compared to the negative control group confirms that the nephrotic syndrome model rats used in this study effectively reflect the pathophysiology of nephrotic syndrome, which involves podocyte dysfunction, the loss of glomerular structure, and increased glomerular membrane permeability [4,42,43,44,45].

In the pathogenesis of nephrotic syndrome, B cells and B-cell-activating factor (BAFF) play critical roles. The initial damage in idiopathic nephrotic syndrome occurs in podocytes, the visceral epithelial cells of the glomerulus. Molecular disruptions in podocyte slit filtration impair the integrity of the glomerular filtration barrier, compromising the filtration of molecules based on size and charge [43,46]. In nephrotic syndrome, B-cell activation mediated by BAFF can trigger the production of inflammatory mediators and autoantibodies that damage podocyte-associated proteins, resulting in podocytopathy. Damage to podocytes, which serve as the final filtration barrier, leads to increased glomerular permeability and protein loss in urine [23,35,36,46,47]. Therefore, inhibiting B-cell activity through a BAFF blockade is a promising approach to prevent podocytopathy and proteinuria in nephrotic syndrome.

This study found that the administration of the *Physalis angulata* extract significantly reduced proteinuria levels in nephrotic syndrome model rats, supporting the role of B cells in the pathogenesis of nephrotic syndrome [23,48,49]. *Physalis angulata* is known to contain Withanolides, which not only reduce oxidative stress-induced free radicals but also have immunomodulatory effects that suppress immune cell activation, particularly of B cells [32,33,34]. The immunomodulatory effect of Withanolides can suppress immune cascades involved in the pathogenesis of nephrotic syndrome by causing glomerular damage and podocytopathy [23,35,36]. In a preliminary in silico study conducted by the researchers, Withanolides showed a direct inhibitory effect on BAFF and its receptor BCMA. Additionally, another bioactive compound in *Physalis angulata*, Physalin F, exhibited inhibitory effects on another BAFF receptor known as TACI (Transmembrane Activator and Calcium-modulator and Cytophilin ligand Interactor) [34]. These findings highlight the clinical potential of *Physalis angulata* as an anti-proteinuria agent.

Moreover, the lower proteinuria levels observed in the combination groups of *Physalis angulata* + prednisone suggest the potential synergistic effects of *Physalis angulata* with prednisone, which remains the first-line therapy for nephrotic syndrome. The most pronounced reduction in proteinuria was observed in the groups receiving *Physalis angulata* at doses of 1500 mg/kg BW + prednisone and 2500 mg/kg BW + prednisone, indicating a potential dose-dependent effect of *Physalis angulata*.

### 4.4. Protective Effects of Physalis angulata

#### 4.4.1. Nephrin in Renal Tissue and Urine

Nephrin, a key protein in the podocyte slit diaphragm, plays a crucial role in maintaining the actin cytoskeleton’s integrity and ensuring the proper function of the glomerular filtration barrier. Reduced nephrin expression in renal tissue in the nephrotic syndrome model is consistent with studies that identify nephrin as a marker for glomerular integrity. Its decline is linked to podocyte loss, disease progression, and proteinuria, indicating early glomerular injury [50]. Furthermore, nephrin becomes a target for pathogenic antibodies such as IgG anti-nephrin in nephrotic syndrome, exacerbating its damage [51,52].

This nephrin damage is reflected in increased urinary nephrin levels, which serve as sensitive early biomarkers of glomerular injury, with a sensitivity of 89% and specificity of 72% [7]. Elevated urinary nephrin levels in the nephrotic syndrome model suggest podocyte injury and the shedding of nephrin into the urine. This phenomenon is strongly associated with proteinuria and declining kidney function. Notably, urinary nephrin is also a more sensitive marker of diabetic nephropathy than microalbuminuria [9,53,54].

Treatment with *Physalis angulata*, alone or in combination with prednisone, showed a renoprotective effect by increasing renal nephrin expression and reducing urinary nephrin levels. The highest level of renal nephrin expression was observed in groups receiving *Physalis angulata* at doses of 1500 mg/kg BW + prednisone and 2500 mg/kg BW + prednisone, suggesting a dose-dependent protective effect. The mechanism likely involves BAFF inhibition, as evidenced by a significant negative correlation between renal BAFF expression and nephrin levels. BAFF inhibition reduces inflammatory cell infiltration, particularly B cells, in the kidney, thereby lowering the pathogenic antibody production that contributes to nephrin damage [34,55].

In urine, *Physalis angulata* treatment reduced nephrin shedding, with the most significant decrease in the group receiving 2500 mg/kg BW + prednisone. This highlights the synergistic effect of *Physalis angulata* and prednisone in stabilizing podocyte structure and mitigating nephrin loss. By preserving the actin cytoskeleton and maintaining slit diaphragm integrity, *Physalis angulata* prevents protein leakage and podocyte injury.

Interestingly, no significant correlation was found between renal nephrin expression and urinary nephrin levels in this study. This may be due to the complex mechanisms of podocyte injury and repair, where structural improvements in podocytes or reduced inflammation may not immediately translate into proportional changes in urinary nephrin excretion. Additionally, podocyte regeneration and filtration dynamics are not always aligned, further complicating this relationship. Despite this, the significant reduction in urinary nephrin levels following *Physalis angulata* treatment suggests a direct impact on glomerular filtration mechanisms, reinforcing its renoprotective role [50,56].

#### 4.4.2. Podocalyxin in Renal Tissue and Urine

A significant decrease in renal podocalyxin expression was observed in the nephrotic syndrome model group compared to the negative control group, indicating the role of podocalyxin as a marker of podocytopathy in nephrotic syndrome. Podocalyxin is a specific protein marker for podocytes, and its urinary levels are significantly elevated in children with primary nephrotic syndrome compared to control groups or patients in remission. Additionally, a positive correlation was found between urinary podocalyxin levels and 24 h urinary protein in patients with acute primary nephrotic syndrome [10]. Podocytes detected in the urine are derived from podocytes that detach from renal tissue, and podocalyxin levels are associated with podocyte injury. This suggests that the quantitative detection of urinary podocalyxin could be useful for evaluating dynamic changes in podocytes and aiding in the diagnosis of nephrotic syndrome. A study by Liu et al. (2012) reported that urinary podocalyxin has a sensitivity of 94.1% for primary nephrotic syndrome [57].

Treatment with *Physalis angulata* prevented the decrease in renal podocalyxin expression compared to the nephrotic syndrome model group. This effect is likely due to the immunomodulatory properties of withanolides in *Physalis angulata*, which suppress B-cell activity through BAFF inhibition. The inhibition of BAFF reduces inflammatory cell infiltration in the kidney, which in turn decreases the production of pathogenic antibodies and pro-inflammatory cytokines that can cause podocalyxin damage [35,36]. This is supported by the significant negative correlation between renal podocalyxin expression and IL-4 levels in this study. The highest podocalyxin expression was observed in the *Physalis angulata* group, at a dose of 2500 mg/kg BW + prednisone, indicating a dose-dependent effect and potential synergy with prednisone, as this group showed significantly higher expression than the prednisone-only group. As a transmembrane protein on the podocyte surface, podocalyxin interacts with the actin cytoskeleton to maintain a normal podocyte structure. This study suggests that *Physalis angulata* preserves the interaction between podocalyxin and the actin cytoskeleton, preventing podocyte damage due to loss of actin integrity [51,54].

A significant increase in urinary podocalyxin levels was observed in the nephrotic syndrome model group compared to the negative control group, consistent with the role of podocalyxin as a marker of podocyte damage. Elevated urinary podocalyxin levels are associated with podocytopathy in various kidney diseases [54]. As a marker of podocyte damage, elevated urinary podocalyxin levels often indicate renal dysfunction, especially in nephrotic syndrome, which is characterized by glomerular structural impairment [5,54,58].

The urinary podocalyxin levels in the treatment group also showed a significant decrease in the *Physalis angulata* 2500 mg/kg BW + prednisone group. Podocalyxin is known to be a marker of podocytopathy and can indicate advanced damage related to the negative charge of podocytes. Podocytes found in the urine represent derivatives of podocytes that detach from renal tissue, and urinary podocalyxin levels correlate with podocyte injury. This study shows that treatment with *Physalis angulata* reduces podocyte damage and prevents the release of renal podocalyxin, as evidenced by the reduced podocalyxin derivatives in the urine.

#### 4.4.3. GLEPP-1 in Renal Tissue

The results of this study show a significant decrease in the expression of GLEPP-1 in the kidneys of the nephrotic syndrome model rats compared to the negative control group. Glomerular epithelial protein 1 (GLEPP-1) is a protein produced by podocytes, the renal epithelial cells that play an important role in maintaining the structure and function of the glomerulus. Severe or persistent podocyte injury can lead to cell detachment, which is the final stage of podocyte injury, causing further glomerular damage. GLEPP-1 is a marker of mature podocytes and is widely used in glomerular diseases. The decrease in GLEPP-1 expression is generally associated with podocyte structural damage. The study used GLEPP-1 to measure podocyte density in Alport Syndrome. In IgA nephropathy, GLEPP-1 expression is known to decrease and is associated with higher pathological levels. GLEPP-1 expression is also known to be lost in patients with lupus nephritis. In cases of Focal Segmental Glomerulosclerosis (FSGS), GLEPP-1 undergoes downregulation. This molecule helps to regulate the pressure and filtration rate, and so the downregulation of GLEPP-1 impacts kidney hyperfiltration [59].

Treatment with *Physalis angulata* in the experimental group improved GLEPP-1 expression by alleviating podocyte damage and enhancing its function in maintaining the glomerular structure. This is supported by previous studies showing that natural substances like *Physalis angulata* can stimulate structural repair in the kidneys through anti-inflammatory and antioxidant mechanisms. The phytosteroid effects of withanolides, which can suppress B-cell activity, also reduce the production of pathogenic antibodies and pro-inflammatory cytokines that can damage podocytes, thus preventing further reduction in GLEPP-1 due to podocyte damage [14,23,35,36,60].

In this study, the combination of *Physalis angulata* with prednisone showed a greater increase in GLEPP-1 expression compared to prednisone alone. Prednisone, as a glucocorticoid, when combined with *Physalis angulata*, which has phytosteroid effects, is likely to produce a synergistic effect that enhances structural and functional kidney repair more optimally [6,14].

## 5. Conclusions

*Physalis angulata* extract significantly improves podocyte integrity and reduces proteinuria in a rat model of nephrotic syndrome through BAFF inhibition. Its combination with prednisone offers enhanced therapeutic benefits, supporting its potential as an adjunct therapy. Future studies should aim to translate these findings into clinical applications, focusing on long-term safety and efficacy.

## Data Availability

The data presented in this study are available on request from the corresponding author due to (specify the reason for the restriction).

## Abbreviations

The following abbreviations are used in this manuscript:

BAFF	B-cell-activating factor
BCMA	Protein Maturation of B Cells
DAMPs	danger-associated molecular patterns
IL-4	Interleukin-4
GLEPP-1	glomerular epithelial protein 1
ELISA	Enzyme-Linked Immunosorbent Assay
FSGS	Focal Segmental Glomerulosclerosis
PAMPs	pathogen-associated molecular patterns
TACI	Transmembrane Activator and CAML Interactor
